# Endogenous Chemiluminescence from Germinating Arabidopsis Thaliana Seeds

**DOI:** 10.1038/s41598-018-34485-6

**Published:** 2018-11-01

**Authors:** Homa Saeidfirozeh, Azizollah Shafiekhani, Michal Cifra, Amir Ali Masoudi

**Affiliations:** 1 Department of Physics, Alzahra University, Tehran, 1993891167 Iran; 2 Institute for Research in Fundamental Sciences (IPM), School of Physics, P.O. Box 19395-5531, Tehran, Iran; 3 Institute of Photonics and Electronics of the Czech Academy of Sciences, Prague, 18200 Czechia

## Abstract

It is well known that all biological systems which undergo oxidative metabolism or oxidative stress generate a small amount of light. Since the origin of excited states producing this light is generally accepted to come from chemical reactions, the term endogenous biological chemiluminescence is appropriate. Apart from biomedicine, this phenomenon has potential applications also in plant biology and agriculture like monitoring the germination rate of seeds. While chemiluminescence capability to monitor germination has been measured on multiple agriculturally relevant plants, the standard model plant *Arabidopsis thaliana* has not been analyzed for this process so far. To fill in this gap, we demonstrate here on *A. thaliana* that the intensity of endogenous chemiluminescence increases during the germination stage. We showed that the chemiluminescence intensity increases since the second day of germination, but reaches a plateau on the third day, in contrast to other plants germinating from larger seeds studied so far. We also showed that intensity increases after topical application of hydrogen peroxide in a dose-dependent manner. Further, we demonstrated that the entropy of the chemiluminescence time series is similar to random Poisson signals. Our results support a notion that metabolism and oxidative reactions are underlying processes which generate endogenous biological chemiluminescence. Our findings contribute to novel methods for non-invasive and label-free sensing of oxidative processes in plant biology and agriculture.

## Introduction

**Figure 1:**
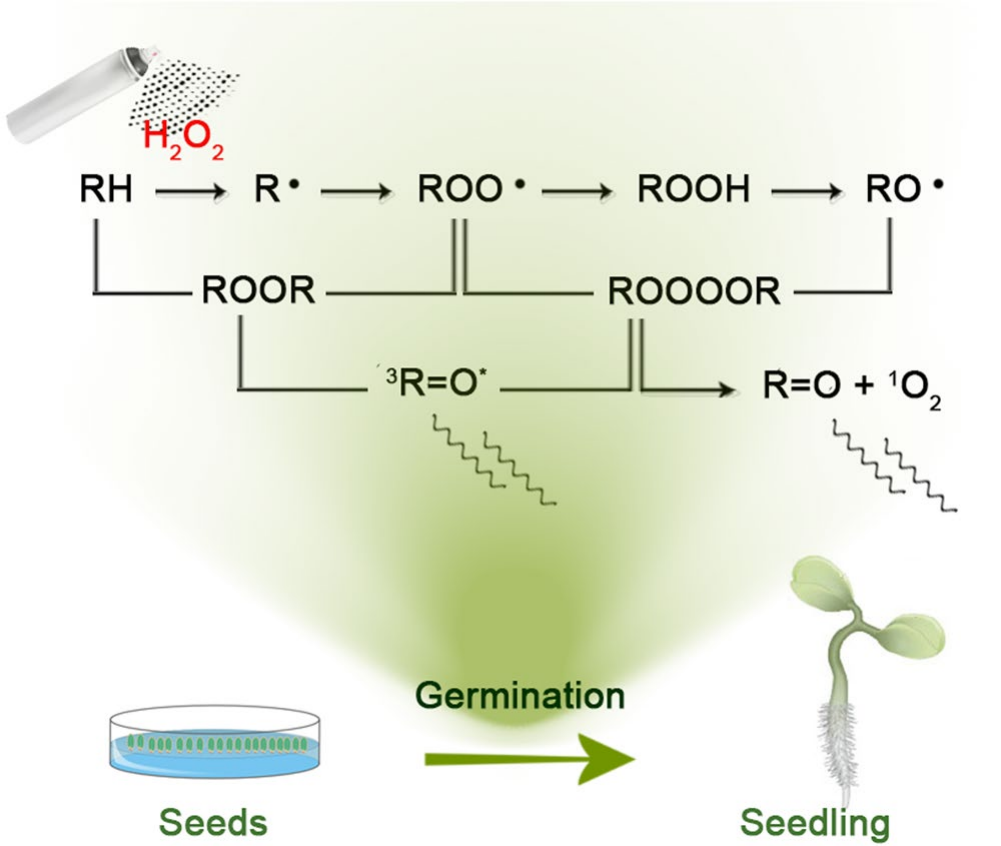
Schematic illustration on the mechanism of endogenous biological chemiluminescence during germination of a seedling. Hydroxyl radical can oxidize all types of biomolecules such as nucleic acids, lipids, and proteins to produce initial radicals of biomolecules. Other oxidation processes can also form biomolecule radicals. During the further reactions, high-energy intermediates such as dioxetane (ROOR) and tetroxide (ROOOOR) can be generated. This high-energy intermediates can be decomposed to electronically excited species such as triplet excited carbonyl or singlet oxygen. Electronically excited species can further react, emit a photon or (in case of triplet excited carbonyl) transfer the excitation energy to a donor - another chromophore which can serve as an emitter at longer wavelengths. Reaction scheme adopted from^2^.

Biological systems continuously generate a light (visible band electromagnetic radiation) of ultra-weak intensity^1^. The currently accepted generating mechanism of this light is as follows^2^. Organisms endogenously produce a small amount of reactive oxygen species (ROS) in the course of their normal metabolism and in an enhanced manner when they undergo stress. A reaction of ROS with biomolecules leads to the formation of, apart from other products, unstable biomolecular intermediates such as dioxetanes and tetraoxides which can produce excited electron species when decomposed. The primary electron excited species considered to be generated are the triplet excited carbonyls and singlet oxygen. Excitation energy can also be transferred to energy acceptor to produce secondary electron excited species. If not deactivated or quenched, these excited states decay radiatively via emission of photons. Therefore, although through a chain of events, the intensity of photon emission reflects a rate of oxidative processes ongoing in the organism (Fig. 1). This connection suggests that detection and analysis of the endogenous biological chemiluminescence (EBC) could be used to monitor oxidative processes in a non-invasive and label-free manner^1^.

Majority of investigations on EBC, which appears in the literature under a variety of names such as ultra-weak photon emission^1^, biophoton emission or autoluminescence^3^, is focused on microorganisms and organisms from animal kingdom reaching to a human with potential relevance in biomedical research and applications. However, a great potential of EBC is also in plant biology and agriculture^4,5^.

A model organism in plant biology is *A. thaliana* (rockcress or thale cress). Botanists and biologists began to research *A. thaliana* in the early 1900s, and the first systematic description of mutants was done around 1945^6^. *A. thaliana* is now widely used in plant sciences^7^, including genetics^8^, evolution^9^, population genetics^10^, and plant development^11^. Although *A. thaliana* has a little direct significance for agriculture, it has several traits that make it a useful model for understanding the genetic, cellular, and molecular biology of flowering plants.

EBC of *A. thaliana* has been explored in several papers. Havaux *et al*.^3^ showed that mutant plants deficient in antioxidants display higher EBC intensity than wild-type plants when treated with light stress. Hence, their experiments confirmed the involvement of ROS in the generation of EBC. Benet *et al*.^12^ observed a burst of photon emission after infiltration of a plant with the avirulent pathogen and demonstrated that the burst requires increases in cytosolic [Ca^2+^] but surprisingly not burst of ROS. Pospíšil and Rastogi^13,14^ employed UV-A and visible light stress on *A. thaliana* and showed that EBC intensity is higher in plants undergoing higher oxidative stress. They also demonstrated the presence of singlet oxygen in isolated chloroplasts using spin-trapping electron paramagnetic resonance spectroscopy. The germination is an important stage of a plant life cycle with tightly regulated growth and metabolism. It would be interesting to employ EBC to probe total rate of oxidative metabolism in germinating *A. thaliana* seeds. However, the dynamics of EBC during seed germination have not been yet explored in *A. thaliana*. So far, past reports explored EBC during germination in agriculturally relevant plants such as wheat^15,16^, rice^17^ and mung beans^18,19^. To fill in the gap, we report in this paper on *A. thaliana* EBC during seed germination and also analyze an effect of externally induced oxidation.

Several authors suggested that statistical properties of EBC time series could carry a useful information about the biological system^20–22^. Discovery of nontrivial statistical properties of EBC could have an impact on understanding of the EBC generating mechanisms^1,23^ and potential role of EBC in optical biological communication^24–27^. A standard approach to analyze statistical properties of the photon signals comes from quantum optics theorems and is focused on photocount statistics of detected light^28^. Employing this approach some authors claimed that EBC manifests quantum optical coherent properties^23^ or even interpreted the observed photocount statistics in terms of quantum optical squeezed states^29,30^. However, the current experimental evidence of such quantum optical properties has been recently criticized^31^. It was pointed out that either meticulous and careful photonic signal preprocessing or employment of well-defined control signals^19^ is needed to avoid artifactual results or misinterpretation of the results. Given the current state of the experimental evidence, the hypothesis that biological photon emission comes from a coherent field seems unlikely to be true. However, it is reasonable to assume that chemiluminescence from chemical and biological systems (as a special case of complex biochemical systems) could exhibit a correlated and complex statistical behavior^32^ which could be analyzed using appropriate fractal or chaos-based methods. Therefore, more recent efforts in the analysis of EBC statistical properties were focused on the various measures quantifying the complexity and correlations in the time series such as Hurst exponent^19^ and multifractal spectra^33^. While EBC statistical properties were already analyzed for germinating seeds of various plants such as cucumber (*Cucumis sp*.), wheat (*Triticum aestivum*)^34^ and mung beans (*Vigna radiata*)^18,19^, no data on statistical properties of *A. thaliana* EBC signals are available so far. In this paper, we focused on the analysis of EBC time series using an Approximate Entropy (ApEn)^35,36^. ApEn has been widely used to assess the level of complexity^37^ and quantifies the regularity or predictability of a time series^38–40^. Furthermore, during the past year’s there has been a rapid rise in the use of ApEn to find the order of dynamical changes in biological systems^41–43^. Large body of literature indicates that ApEn is a useful measure to evaluate dynamical evolution in non-linear and non-stationary signals such as electroencephalography^44,45^, electromyogram^46^ and electrocardiogram^47^.

## Results and Discussion

### Endogenous biological chemiluminescence during germination

**Figure 2:**
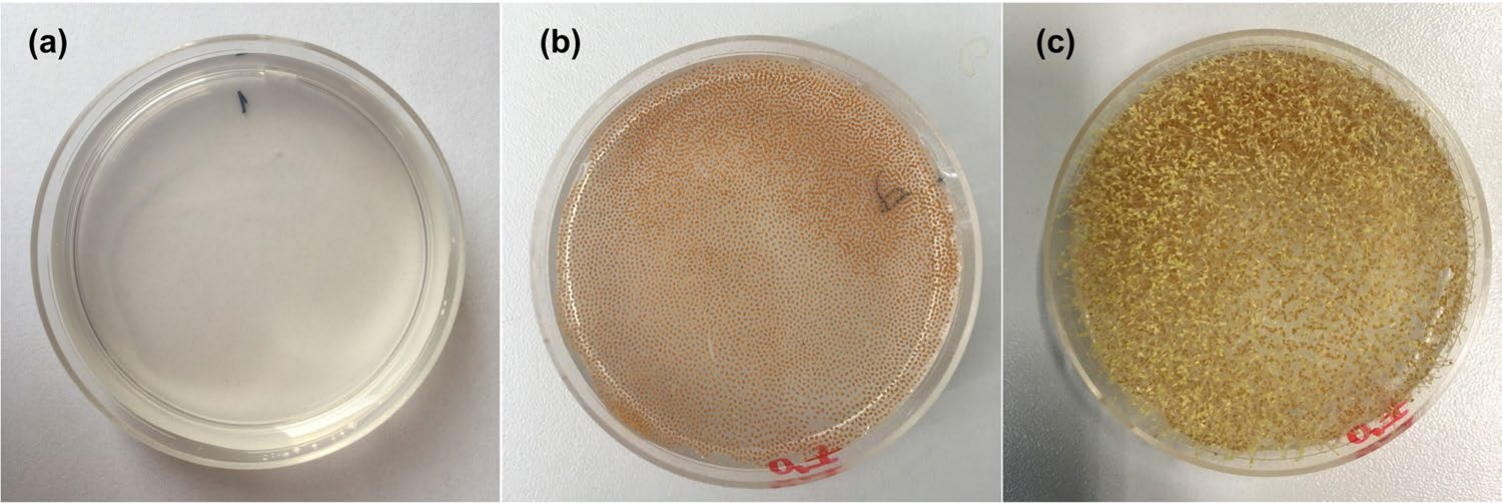
(**a**) Agar dish, (**b**) *A. thaliana* seeds on agar dish just after deposition, (**c**) *A. thaliana* seeds germinated on agar dish after three days.

**Figure 3:**
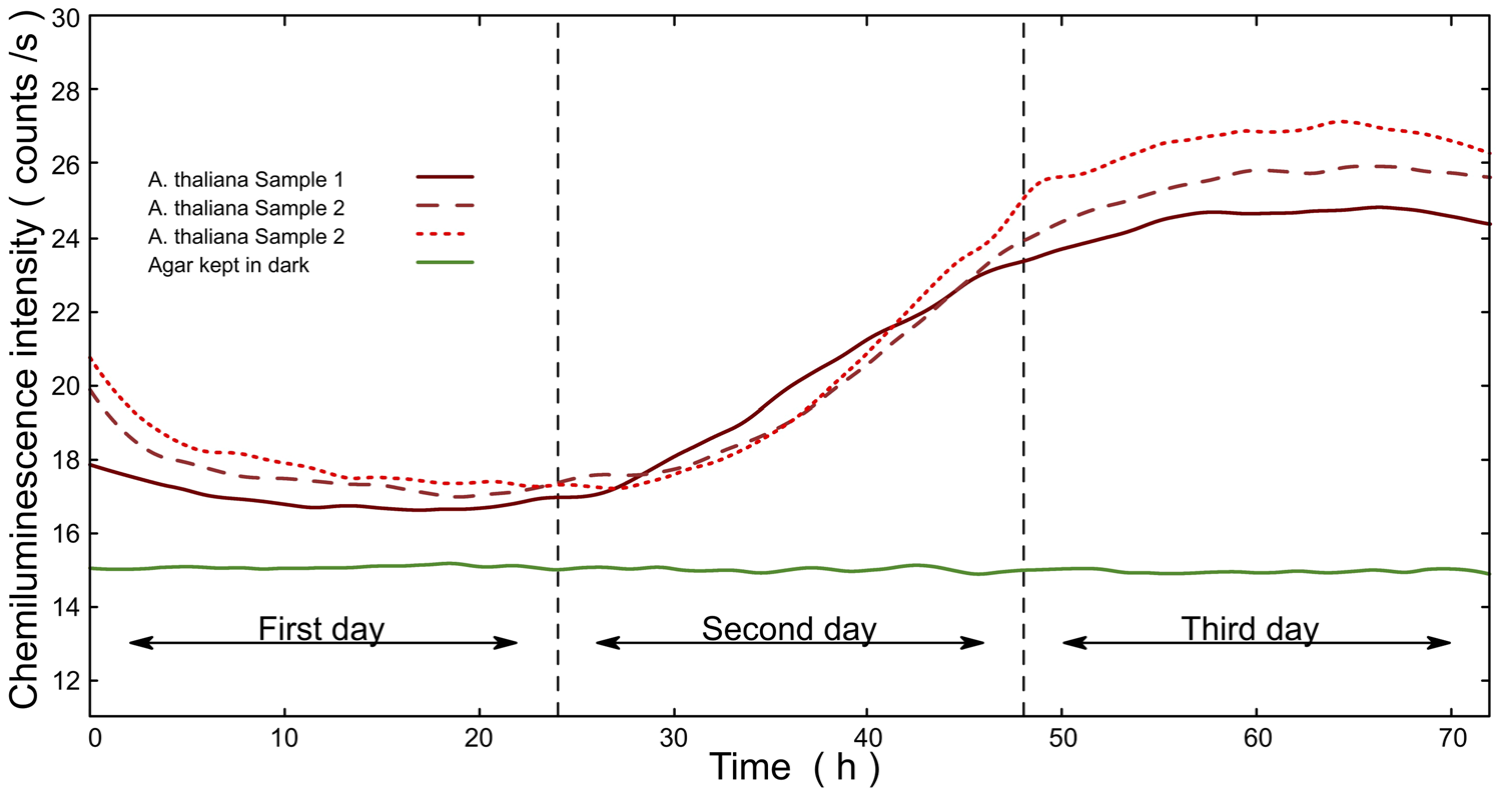
Red lines represent endogenous biological chemiluminescence from germinating *A. thaliana* seedlings samples. Green line represents signal from agar. The lines are produced from the raw data using smoothed LOESS algorithm. Note that the photodetector noise (mean counts/s = 12.5) is included in the signals.

To study the EBC during the germinating *A. thaliana*, we measured EBC signals from *A. thaliana* seedling for three consecutive days (Fig. 3). As the germination as well as measurement was done in the light tight black chamber, emerging leave tissue was white under these etiolating conditions (Fig. 2(c)). Roughly 85% of seeds germinated. All the measurements were taken using the low-noise PMT. Results show (Fig. 3) that the signal during the first day displayed a weak delayed luminescence probably as a consequence of treating the seeds with 24 hours of light (See Materials and Methods). Interestingly, from the second day, the EBC intensity started to increase (Fig. 3). We believe that the increase of the signal intensity is related to an increase of the total plant tissue volume undergoing (oxidative) metabolism, following the working hypothesis: the higher the volume and surface area of the sample, the more EBC signal emitted from the sample. Our results are corroborated by earlier works on EBC detection and plant germination: a correlation between EBC intensity and germination process was already demonstrated in our previous work where germinating mung beans displayed a continuously increasing signal over six days of germination^18^. Other authors showed that plants such as wheat also displayed a slow increase of EBC intensity during 6 days germination^48^. However, there are notable quantitative and qualitative differences in our data compared to these earlier works. While in the case of germinating mung beans^18^ and wheat^48^ the EBC intensity tends to increase steadily during the whole period of measurement (5-6 days), we observed that EBC from *A. thaliana* ceases to grow at the day 3 in our conditions. The EBC dynamics may be explained by natural patterns of seedling growth (hence metabolism) under the given experimental conditions. As the experiments were conducted under “hungry” conditions (no nutrients in agar, only water) and no light for photosynthesis, the seed growth and germination depends on the resources stored in the seed only. *A. thaliana* seed has a size (expressed via projection surface) only about 0.1 mm^2 ^^49^, Wheat and mung beans have seed surface of about tens of mm^2^. Large seeds provide more resources and consequently enable longer period of growth without a need of an external supply of nutrients and also provide a greater ability to tolerate stresses than the small seedlings^50^. The amount of stored resources per seed might be an explanation of the different growth dynamics of larger seeds vs. small *A. thaliana* seeds we observed here. In earlier works on wheat germination, it was also showed that the increasing trend of the EBC signal intensity might not be monotonous but may undergo fluctuations on the time scales of hours which were suggested to be related to gravimetric changes^51–53^. In contrast to those findings, we did not observe any notable fluctuations and oscillations of the EBC trend in our *A. thaliana* data. Since the cause of the fluctuations observed in^51–53^ are not fully elucidated, we will not engage in discussion why we did not observe them in our data. Overall, our data seem to support the hypothesis that EBC is correlated with cellular metabolism.

### Endogenous biological chemiluminescence after treatment with H_2_O_2_

**Figure 4:**
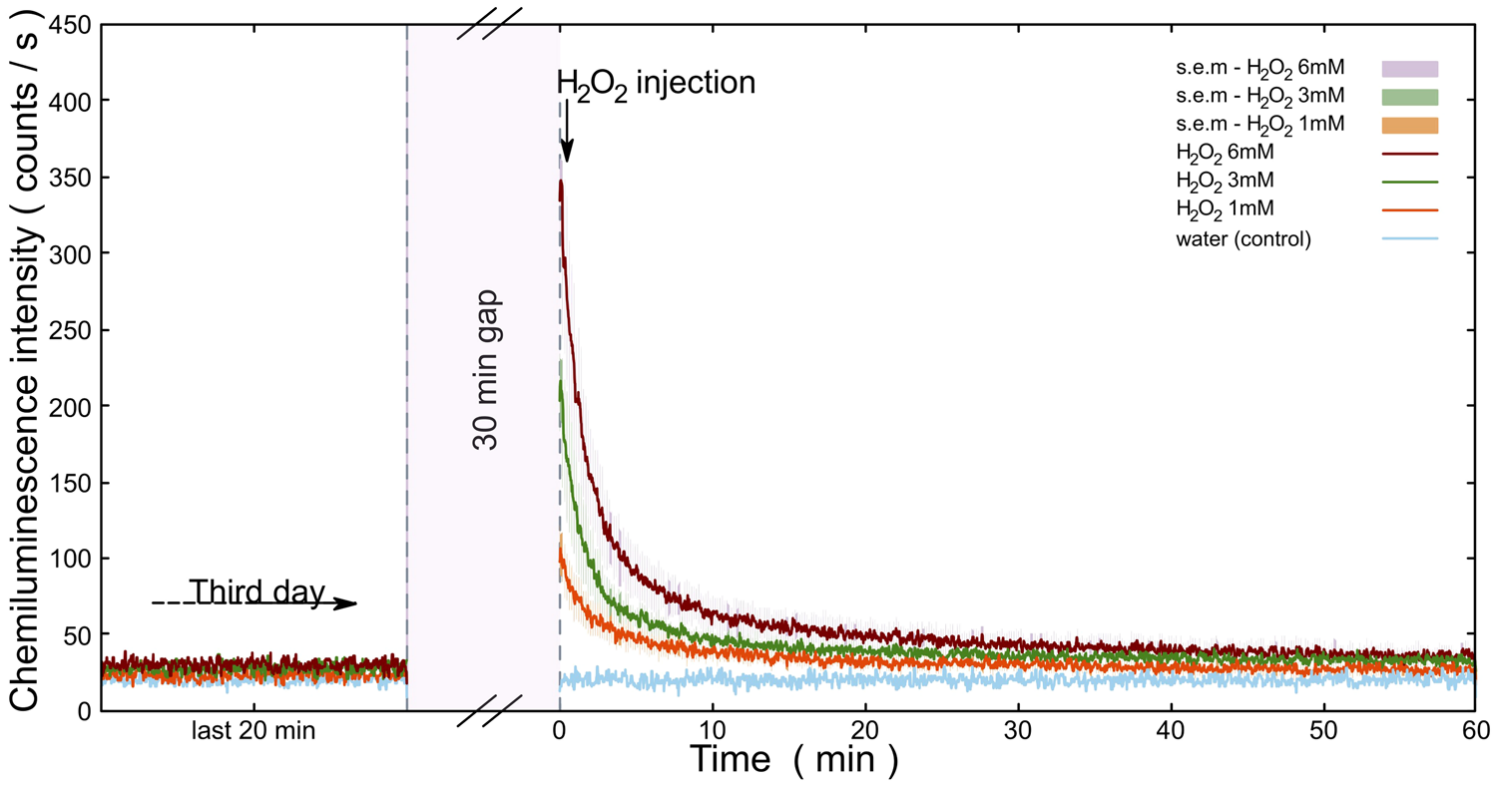
Hydrogen peroxide (H_2_O_2_) increases intensity of endogenous chemiluminescence of *A. thaliana* seedlings in a dose-dependent manner. H_2_O_2_ was applied on germinating seeds after three days after a deposition on agar plate. There was always 60 s gap between injection of H_2_O_2_ and start of the measurement due to sample manipulation. Standard error of mean is calculated for n = 3 measurements.

**Figure 5:**
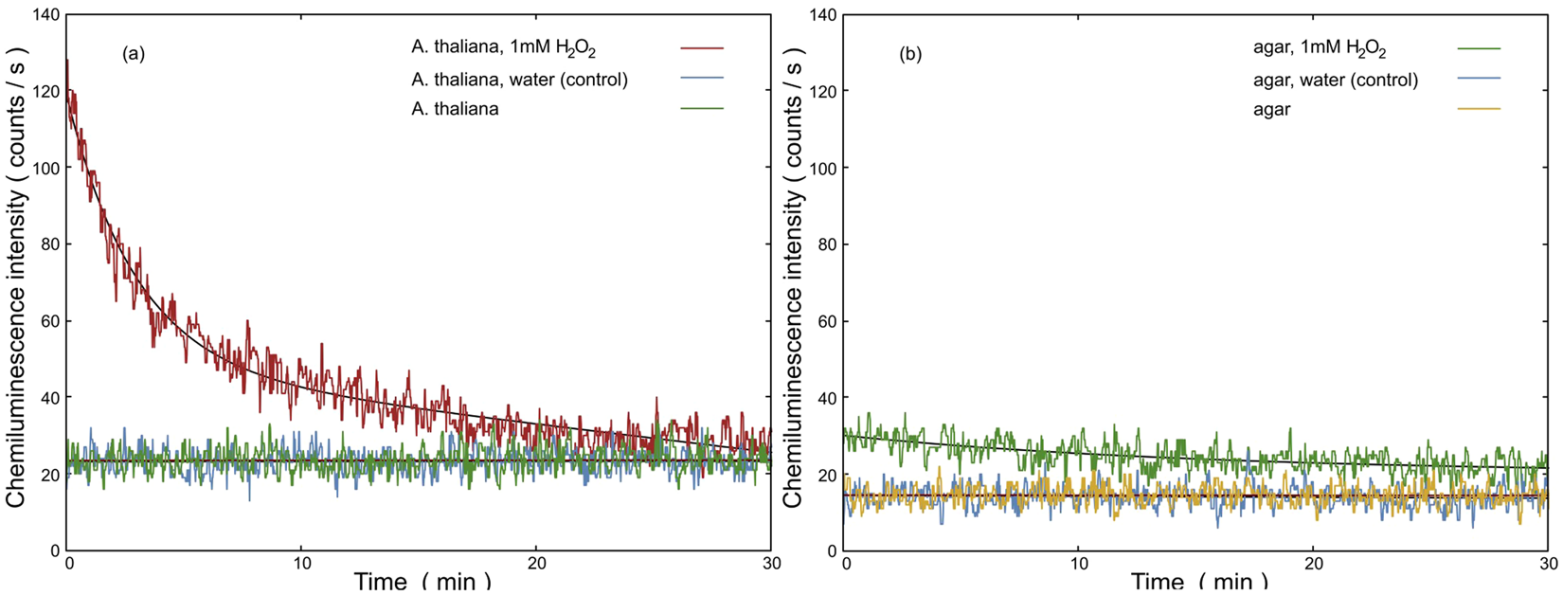
(**a**) and (**b**) compare the effect of spraying 100 *μ*L hydrogen peroxide (1 mM) and water (as a control treatment) on the chemiluminescence from *A. thaliana* seedlings and agar autochemiluminescence, respectively. The smooth solid lines show the best fit of the data using a polynomial function. The chemiluminescence measurement started at 30 minutes after completion of the test on the third day, and it was measured for 30 minutes using a photomultiplier detector. Samples were kept in the dark for three days before treatment and measurement. There was always a 60 s gap between injection of either H_2_O_2_ or water and the start of the measurement due to sample manipulation.

The current paradigm of EBC generating a mechanism (see Fig. 1 and^2^) predicts that the higher the rate of biomolecule oxidation the higher EBC intensity. In order to test if this hypothesis holds in our system, we sprayed H_2_O_2_ on the *A. thaliana*. We assume that metal ions in a reduced state (such as Fe^2+^ or Cu^+^ and others) endogenously present in the plant tissue mediate formation of hydroxyl radical, a strong oxidant, from H_2_O_2_ via Fenton reaction^2^. Hence, the addition of H_2_O_2_ should lead to a transiently increased oxidation rate of biomolecules and consequently to a transiently increased EBC intensity from biosample. For H_2_O_2_ treatment of *A. thaliana* samples, we sprayed 100 *μ*L H_2_O_2_ at different concentration as follows: 1 mM, 3 mM and 6 mM. The results can be seen in Fig. 4. Each treatment was also reproduced in triplicates (Figure S3). We can see from the Figure S3 that the impact of H_2_O_2_ treatment is rather reproducible. It can be observed that H_2_O_2_ increases intensity of EBC in a dose-dependent manner (Fig. 4). After the treatment, the trend of intensity seems to follow an exponential-like decay. The kinetics of the process chemiluminescence induced by H_2_O_2_ (Fig. 4) should be briefly discussed. The addition of H_2_O_2_ causes initial burst of light, because the Fenton reaction and initial oxidation of biomolecules by hydroxyl radical (see formation of alkyl radical R in Fig. 1) is rapid (rate constant k = 10^5^–10^9^ M^−1^s^−1^)^54^. After initial addition, the H_2_O_2_ and consequent products intermediate to photon emission start to run out and the system tends to return to a steady state - hence the decay (decrease) of chemiluminescence. The time scale of decay we observe corresponds to some rate limiting reaction different from the Fenton reaction and primary oxidation of biomolecules. Control treatment (water sprayed instead of H_2_O_2_) showed that the increase of signal was indeed due to H_2_O_2_ and not due to the spraying itself (Fig. 5). Agar as a substrate for seed germination represents a significant fraction of an optically accessible surface of the sample even after three days of germination (Fig. 2). Therefore, we needed to check how much the agar treated with H_2_O_2_ contributes to the signal after treatment. We found that the small increase of the signal is observable after H_2_O_2_ treatment of agar plate without seeds compared to water control (Fig. 5b). However, the contribution to measured EBC due to agar H_2_O_2_ oxidation is negligible when compared to the EBC signal from *A. thaliana* sample (Fig. 5). Our results suggest that EBC is due to oxidative processes in plant tissue. UV-A induced oxidative stress of *A. thaliana* plants was showed to increase EBC intensity^13,14^. It was also showed^3^ that *A. thaliana* mutants deficient in antioxidants such as ascorbate or zeaxanthin (*vtc2 npq1* single or double deletion mutants) displayed higher EBC intensity when treated by photoinduced oxidative stress. In the potato tuber tissue, endogenous H_2_O_2_ production was correlated to EBC intensity^55^, thus supporting our results that H_2_O_2_ might be involved in EBC generation in the plant tissue.

### Approximate entropy analysis

**Figure 6:**
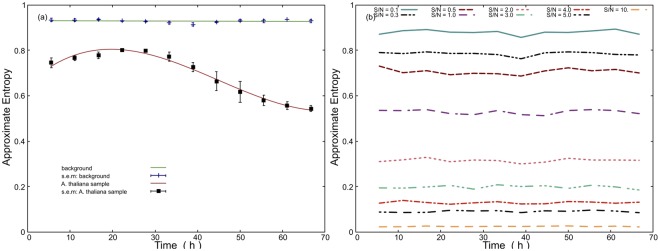
(**a**) The value of approximate entropy is stable for the background (photodetector) noise while evolves for the *A. thaliana* signals. The trend of approximate entropy time dependence is reproducible and is related to signal-to-noise ratio, see Fig. 3, errorbars are from n = 3 measurements. (**b**) Values of approximate entropy depend on the signal-to-noise (S/N) ratio of Poisson signal and photodetector noise. Poisson signals were computer generated with various mean values and added to experimentally obtained photodetector noise to obtain various S/N ratio.

The ApEn method was used to assess the complexity of *A. thaliana* plants EBC signal. Our hypothesis was that ApEn could be used to distinguish between various stages of *A. thaliana* seedling germination and that the values of ApEn of *A. thaliana* are different than the detector noise. The rationale is that EBC originates from a biological system which possesses a certain degree of complexity (dis/order) while detector noise comes from the technical source and is of a thermal origin. To reduce effects of the potential non-stationarity behavior of the signal, we split signals into segments of 20000 seconds (20000 samples). The results are in Fig. 6(a). We see that the ApEn has a stable and high value for the background (essentially photomultiplier detector noise). However, the ApEn value of *A. thaliana* signals displays a certain time-dependence. During the first day, the ApEn value is rather high (0.8) and then starts to decrease, approaching the value of 0.5. Under closer observation, one can see that the trend of ApEn (Fig. 6(a)) seems to be opposite to the trend of EBC intensity (Fig. 3). To interpret this observation, we decided to produce well-defined control signals: we prepared computer generated random Poisson signals (to represent statistical properties of EBC - see Materials and Methods) of various mean values superimposed on the experimentally obtained detector noise. We generated these control signals with various signal-to-noise ratios and then ran ApEn analysis on them. Results are in Fig. 6(b). We see that the higher the S/N ratio, the lower the ApEn value. For the value of ca. S/N = 1 (*i.e*. detector noise value of *ca*. 12.5 cps and pure signal intensity around 13 cps in Fig. 3 (note that the experimental values always include also the detected noise)) the value of ApEn = 0.55 which very accurately matches experimental situation of *A. thaliana* on a third day (Fig. 6(a)). The fact that the control signals based on computer generated random Poisson signals manifest the same value of ApEn as the experimentally detected *A. thaliana* suggest that *A. thaliana* signals also have a random Poisson nature. This result has repercussions to our understanding of the physical nature of the generating process of biological photon emission: it seems that the EBC signal from *A. thaliana* does not carry more information than a computer generated random Poisson signal. This interpretation is in concordance with the idea that EBC is emitted in an uncorrelated process from independent emitters. One should be careful not to overgeneralize our results: our findings are valid for *A. thaliana* germinating seeds and for ApEn. We can not exclude that other biological or biochemical systems will not manifest certain correlations in the chemiluminescence kinetics^32^. Anyway, we strongly recommend to always using well defined (a computer generated) signals including the detector noise as a control for sophisticated signal analysis to avoid misinterpretation of results from the photonic signal analysis. Surrogate signals created by experimental signal randomization might not be the best option for control signals as they do not elucidate the contributions of signal components (for instance detector noise vs. pure signal from the sample) to the observed signal properties. While our findings from the approximate entropy method suggest that the statistical properties of EBC from *A. thaliana* are rather trivial, it is rather surprising that the ApEn method seems to be very sensitive to the presence of even a small amount of Poisson signals added to the photodetector noise (background). For example, even for S/N = 0.5 the ApEn value is substantially different from the background signal (Fig. 6(a)). We suggest that ApEn could be used to sense a presence of even a weak Poisson signal of various origins in the detector noise. Therefore, ApEn could be used as a measure to assess whether the signal detected is due to the increased noise of the photodetector itself or due to an unwanted weak light source present in the dark chamber. One should also be aware that ApEn value of photodetector noise could be dependent on the set gain and discriminator threshold.

### Imaging of endogenous chemiluminescence

**Figure 7:**
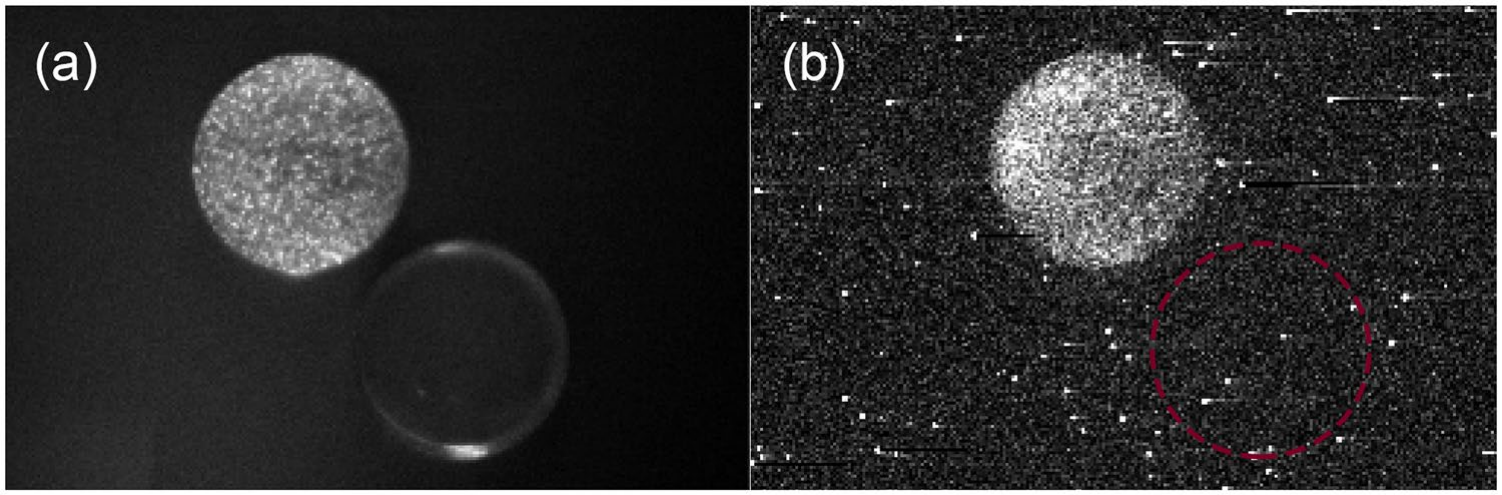
(**a**) A photograph of the *A. thaliana* seedlings and agar taken under weak light illumination. (**b**) Endogenous chemiluminescence from *A. thaliana* seedlings and agar. Imaging was performed using a highly sensitive EM-CCD camera with an integration time of 30 minutes.

**Figure 8:**
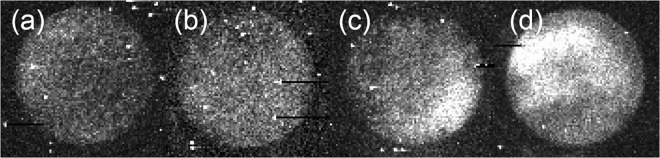
Imaging of chemiluminescence signals from *A. thaliana* seedlings: (**a**) endogenous chemiluminescence with no treatment (control), and after treatment with spraying 250 *μ*L of (**b**) 1 mM H_2_O_2_, (**c**) 3 mM H_2_O_2_ and (**d**) 6 mM H_2_O_2_ Imaging was performed using a highly sensitive EM-CCD camera with an integration time of 30 minutes.

We performed imaging of endogenous chemiluminescence from *Arabidopsis thaliana* samples to obtain insight into the spatial distribution of EBC signal from the sample. Figure 7(a) represents a photograph of the *A. thaliana* plants and agar under weak light illumination. Figure 7(b) shows chemiluminescence of *A. thaliana* seedling sample and agar plate. We can observe that the pattern of chemiluminescence intensity of the *A. thaliana* sample is slightly inhomogeneous with a higher signal on the top and left side of the dish. This finding can be probably due to the inhomogeneous distribution of the germination of seeds: see Fig. 2(c) which displays the color photograph of the same sample. We also tested how the effect of H_2_O_2_ treatment affects the EBC intensity following the same hypothesis as in the section 0. The results can be seen in Fig. 8(a–d). We sprayed 250 *μ*L of H_2_O_2_ with concentration 1 mM, 3 mM and 6 mM on the *A. thaliana* samples. It can be seen that the higher the H_2_O_2_, the higher the chemiluminescence intensity. The apparent lack of homogeneous distribution of chemiluminescence intensity can be attributed to inhomogeneous seeds germination and inhomogeneous spraying as well. Despite the limitations of inhomogeneous germination and spraying, our findings do nevertheless suggest that EBC from the sample with the highest concentration of H_2_O_2_ applied (Fig. 8(d)) was considerably higher compared to the EBC from the sample with the lowest H_2_O_2_ concentration (Fig. 8(b)). These results again confirm that H_2_O_2_ and oxidative processes are involved in the generation of EBC.

### Molecules and processes contributing to endogenous chemiluminescence from A. *thaliana*

The EBC is a signal proportional to an integral rate of oxidation in the tissue, here a plant tissue. The question “Oxidation of which type of biomolecule is a major determinant for EBC” is very interesting yet difficult to answer. The first aspect of the answer is a relative abundance of the biomolecules and the second aspect is a yield of chemiluminescence due to biomolecule oxidation. Concerning the biomolecules which are prone to oxidation, *A. thaliana* seeds are rich in lipids (~30%) and proteins (~35%)^56^. The rest of seed mass is water, a small amount of starch and other compounds. During the germination, the storage lipids (fatty acids) and proteins (12 S globulin (cruciferin) and a 2 S albumin (arabin))^56^ are used as a source of carbon and nitrogen, respectively, for the synthesis of new lipids and proteins. Those lipids and proteins, which are most abundant and most accessible to oxidation with an end product being an (excited) carbonyl, contribute most to EBC. It was showed^57^ that the most abundant protein carbonyls during the *A. thaliana* germination are the Rubisco large chain, heat shock protein of 70 kD (Hsp70) chaperones, glycoside hydrolase family 1 proteins, cytosolic lyceraldehyde-3-phosphate dehydrogenase, Fru-1,6-bisphosphate aldolase, aminopeptidases, the mitochondrial F0F1-ATP synthase b-subunit and several translation initiation and elongation factors. While these are the major protein carbonyls found in germinating *A. thaliana*, they may not necessarily be those which contribute most to the EBC. Direct evidence of a specific protein carbonyl being an emitter of EBC or being a second product when singlet oxygen as EBC emitter is produced is difficult even in a pure chemical system not speaking about a complex system such as a plant tissue.

## Conclusion

In this paper, we measured endogenous chemiluminescence from *A. thaliana* seeds during germination for the first time. We found that the intensity of chemiluminescence increases during the second day and reaches a plateau on the third day under our conditions. The results are qualitatively consistent with the chemiluminescence data from seed germinating experiments from other plants. However, the novelty is in the finding that the quantitative dynamics of EBC from *A. thaliana* seeds differs from other plants analyzed so far: the EBC intensity reaches a plateau after three days, which was not observed in other plants with larger seeds. Furthermore, we demonstrated that hydrogen peroxide treatment increases chemiluminescence intensity in a dose-dependent manner. The results from chemiluminescence time series measurement and imaging corroborated each other while also provide a complementary aspect. While time series data capture chemiluminescence kinetics in a real-time manner, the imaging includes information on a spatial inhomogeneity of the seed chemiluminescence across the dish which is invisible in the integral photomultiplier-based time-series measurement. As the “information” content of EBC has been subject of discussion and speculations, we also analyzed the information content, or orderliness, of EBC signal using approximate entropy method. Our results suggest that the “orderliness” of EBC signals from *A. thaliana* is the same as that of computer-generated random Poisson signals. However, we also found that the EBC signal entropy is different from the detector noise. Approximate entropy could be used for sensitive detection of Poisson signals presence. We believe that our results contribute to elucidate capabilities of EBC as a completely non-invasive, label-free method for monitoring oxidative metabolism in plants and they can shed new light on the statistical properties of EBC.

## Materials and Methods

### Plant material and growth conditions

The *Arabidopsis thaliana* (Columbia ecotype 0) seeds were used in experiments. At first, the seeds were placed to a distilled water for 48 hours at 4 °C and then placed under light for 24 hours (internal light of the flowbox ESCO Airstream Class II BSC) to initiate germination processes. To germinate the seeds, Petri dishes with 1.2% agar medium without Murashige and Skoog salts or sucrose, pH 5.8, have been prepared (Fig. 2(a)).

To measure only endogenous chemiluminescence, we needed to avoid delayed luminescence (combination of unspecific phosphorescence and light-induced chemical reactions which might generate the electron excited states and consecutive photon emission). Therefore, we kept agar plates in dark conditions for at least 10 days, while 3 days seem to be the minimum needed to get rid of any delayed luminescence (Figure S2). Around 7000 seeds were deposited evenly on a 6 cm diameter agar dish (Fig. 2(b)). The measurement of chemiluminescence from the seeds then started and was performed for 3 days in the light-tight chamber (custom-made by the Bioelectrodynamics research team, Institute of Photonics and Electronics of the Czech Academy of Sciences). The air with the temperature of 22 °C in the chamber was saturated with water vapor using an open beaker with water to prevent drying of the agar.

### H_2_O_2_ Treatment

In the H_2_O_2_ treatment, the *A. thaliana* seedlings were sprayed with freshly prepared 1 mM, 3 mM and 6 mM hydrogen peroxide (from stock solution ~30% w/v in water, source Ing. Petr Švec - PENTA s.r.o.). Hydrogen peroxide was diluted in mili-Q water for preparing the 1 mM, 3 mM, and 6 mM concentration. Furthermore, a water control (i.e., 0 mM) sample was prepared. All the plant and agar samples were kept in the dark, light-tight chamber for three days at 22 °C before H_2_O_2_ treatment. For a photomultiplier measurement (chemiluminescence time series) and imaging, 100 *μ*L and 250 *μ*L of H_2_O_2_ was sprayed on the surface of the sample, respectively. Each experiment was repeated at least three times. Vehicle (water) was used as a control.

### Measurement of chemiluminescence time series

For detecting chemiluminescence the photomultiplier tube (PMT) module H7360-01 (Hamamatsu Photonics Deutschland, DE) selected for low noise and counting unit Altera DE2 board (Terasic) with in-house programmed scripts in Matlab were used. The spectral response of the PMT was within the range of 300 nm to 650 nm. PMT module was mounted from the top outer side of the black light-tight chamber (standard black box 41 × 41 × 41 cm^3^ (Figure S1), Institute of Photonics and Electronics, CZ). Dark count of the PMT was *ca*. 12.5 counts/s. The temperature inside the black light-tight chamber was maintained to 22 °C using Peltier Cooling Air to Air 100 W (UWE Elektronik, Germany). The distance between the PMT module input window and the surface of agar was 15 mm (Figure S1).

### Signal processing

In the figures displaying chemiluminescence time series, we display either raw signals or signals smoothed using LOESS (Local regression using weighted linear least squares and a 2^*nd*^ degree polynomial model) method^58^.

### Imaging of chemiluminescence

For imaging of the chemiluminescence from *A. thaliana*, a highly sensitive Electron Multiplying Charged Couple Device (EM-CCD) camera Andor iXon Ultra 888-BV (Andor, UK) and the Andor SOLIS software (v.4.29.30012.0) was used. The EM-CCD chip was cooled to the −95 °C to decrease the thermal noise. The spectral sensitivity range of the EM-CCD camera was 300–1100 nm and the objective used for imaging was Xenon 0.95/25 mm. The EM-CCD camera module was mounted from the top outer side of the black light-tight chamber (standard black box 41 × 41 × 41 cm^3^, Institute of Photonics and Electronics, CZ). For imaging of the *A. thaliana* seedlings and agar were kept at 30 cm away from the EM-CCD camera. The EM-CCD camera parameters were set as follows: gain 1, readout rate 1MHz at 16-bit and exposure time 30 min and a 4 × 4-hardware binning. All images were captured at the resolution of 1024 × 1024 pixels. Before each measurement, all the samples were kept at the light-tight dark chamber for three days.

### Approximate Entropy

Approximate entropy (ApEn) has been introduced as a quantification of regularity or predictability in time series data. It is a method that shows that the trends of the data patterns that are close to each other will remain close in the next measurement with the next pattern. ApEn is obtained with respect to a free parameter r as follows:

1 $$ApEn(m,r,N)={\varphi }^{m}(r)-{\varphi }^{m+1}(r)$$

where ${\varphi }^{m}(r)$ is defined as

2 $${\varphi }^{m}(r)={(N-m+\mathrm{1)}}^{-1}\sum _{i=1}^{N-m+1}{\rm{l}}{\rm{o}}{\rm{g}}{{C}_{i}}^{m}(r)$$

where ${{C}_{i}}^{m}(r)$ is defined as the maximum absolute difference between their respective scalar components of times series^59^. r is defined as 0.2 times the standard deviation (SD) of the data set^60^. In order to carry out ApEn analysis we used ApEn method^61^ and MATLAB software^62^.

As control signals, we generated random Poisson signals with the same length as *A. thaliana* EBC signal using MATLAB *poissrnd*^63^ function. We choose the value *λ* as mean value intensity of EBC signals. This gives a signal with added uncertainty that is Poisson distribution random variable. Then, detector noise is added to the generated random Poisson signals as constant to obtain a control signal. We generated control signals with 9 various signal-to-noise ratios (i.e. $\frac{\lambda }{mean(detector-noise)}=0.1,\,0.3,\,0.5,\,1,\,2,\,3,\,4,\,5\,{\rm{a}}{\rm{n}}{\rm{d}}\,10$) and then run the ApEn analysis on them.

## Electronic supplementary material

Supplementary information

## Data Availability

The raw data are available on request from corresponding authors.
