# Supplementary material for: Endogenous Chemiluminescence from Germinating Arabidopsis Thaliana Seeds
Source: Sci Rep. 2018 Nov 1;8:16231. doi: 10.1038/s41598-018-34485-6 (PMC6212569; doi:10.1038/s41598-018-34485-6)
Supplement: Supplementary file 1 — Supplementary information [file 41598_2018_34485_MOESM1_ESM.pdf]

Supplementary data of the article: ”Endogenous Chemiluminescence from Germinating Arabidopsis Thaliana Seeds”

Homa Saeidfirozeh[1,2], Azizollah Shafiekhani[1,2,*,+], Michal Cifra[3,*,+], and Amir Ali Masoudi[1]

[1]Alzahra University, Physics Department, Tehran, 1993891167, Iran
[2]Institute for Research in Fundamental Sciences (IPM), School of Physics, P.O. Box 19395-5531, Tehran, Iran
[3]Institute of Photonics and Electronics of the Czech Academy of Sciences, Prague, 18200, Czechia
[*]ashafie@alzahra.ac.ir, cifra@ufe.cz
[+]these authors contributed equally to this work

ABSTRACT

Here we provide supplementary information of the article: ”Endogenous Chemiluminescence from Germinating Arabidopsis Thaliana Seeds”.

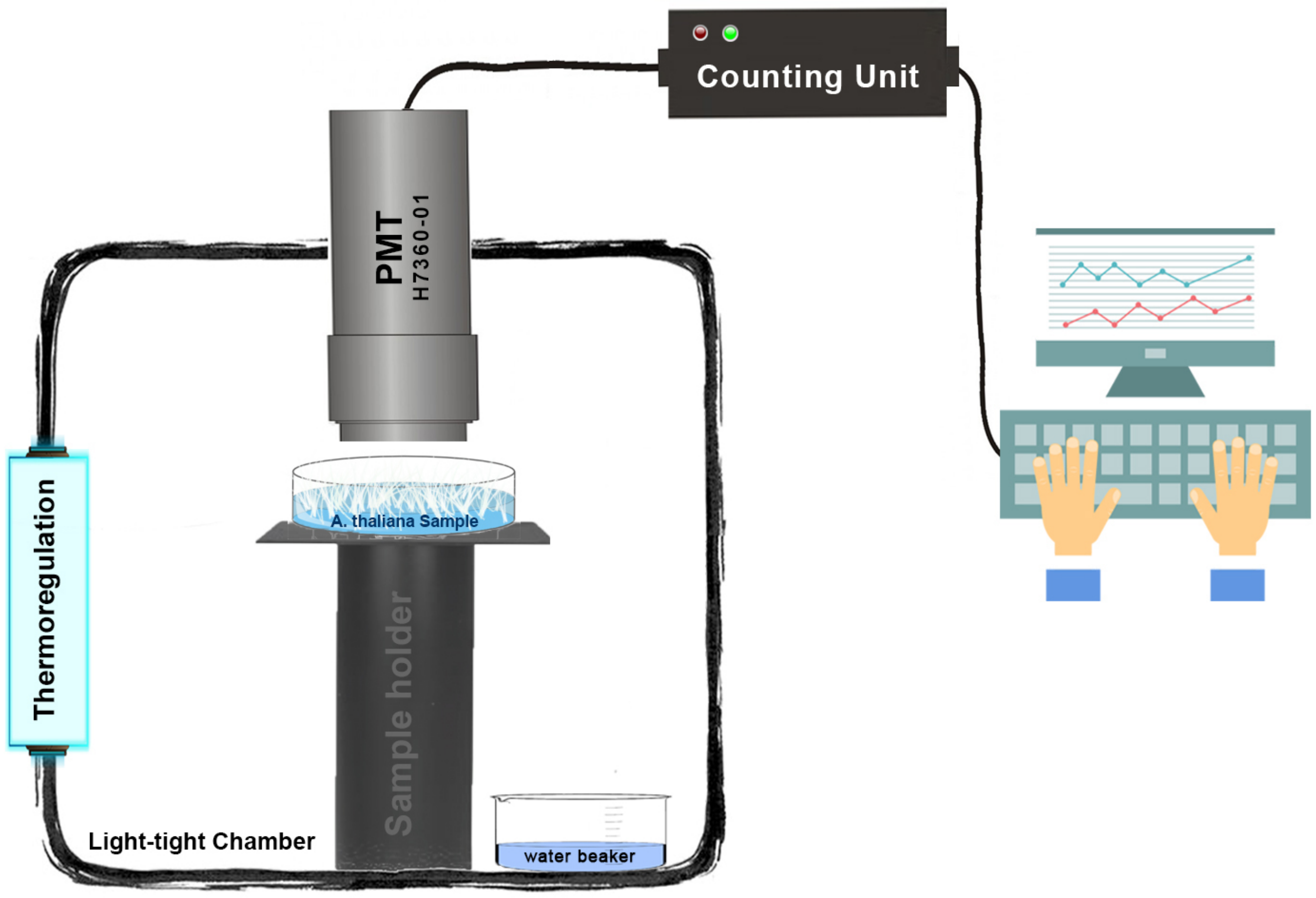

Figure S1. Schematics of the experimental setup for detection of endogenous biological chemiluminescence.

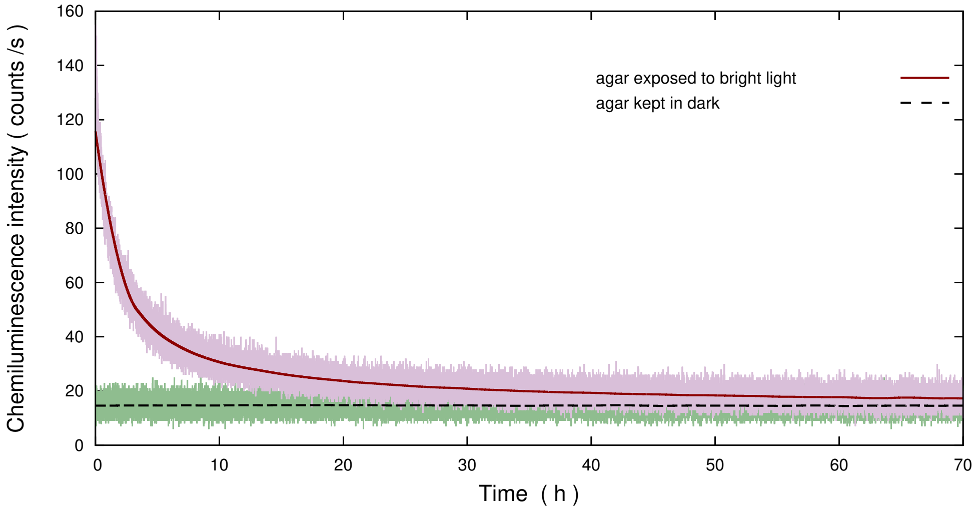

Figure S2. Autochemiluminescence detected from agar dish left in dark for three days and agar dish exposed to flow-box light (see Methods) for 10 hours, respectively.

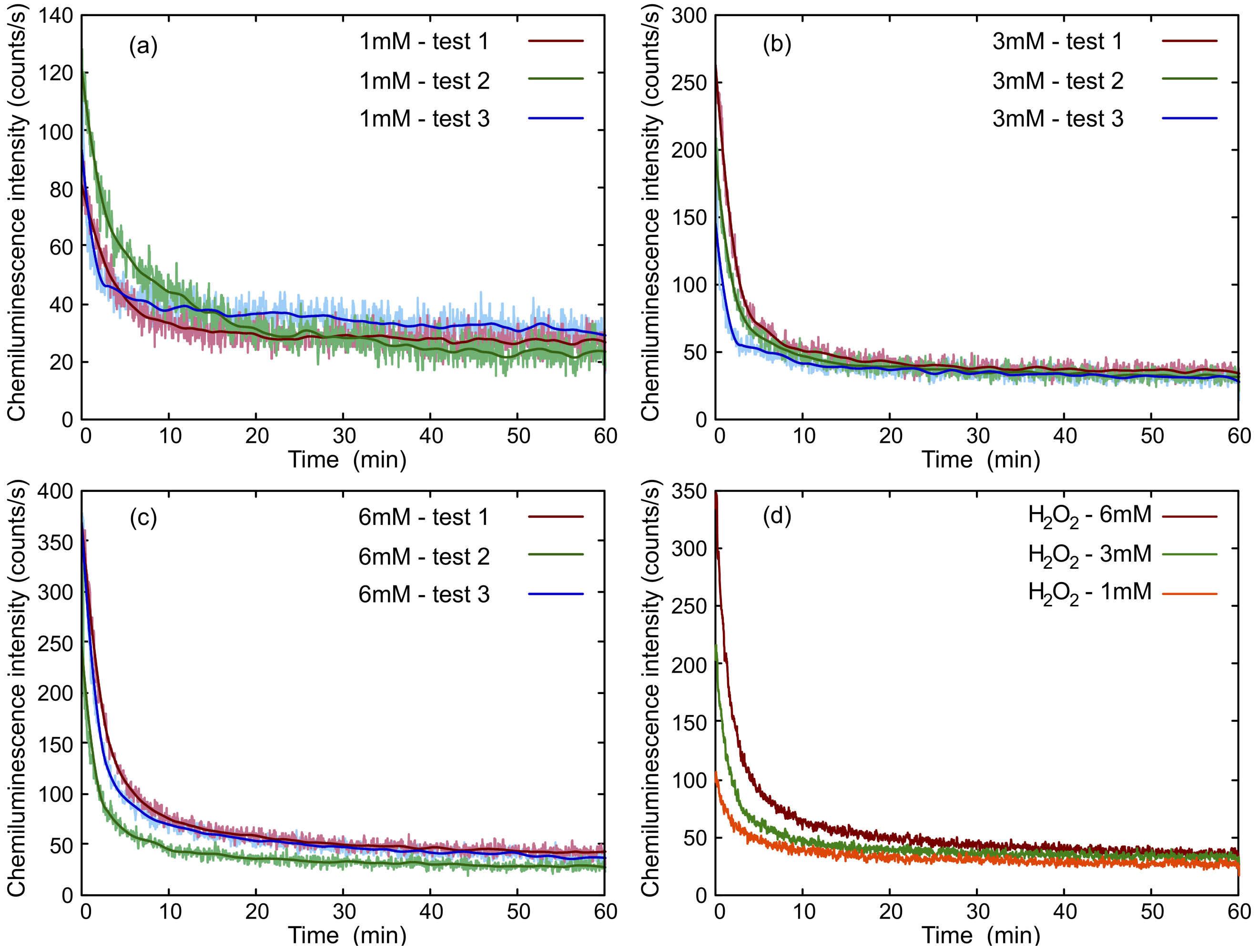

Figure S3. Endogenous chemiluminescence signals from *A. thaliana* after spraying with H_2O_2 are reproducible. (a) - (c) represent different H_2O_2 concentrations 1 mM, 3 mM and 6 mM were used, respectively. All the samples were kept in dark room for three days before measurement. Chemiluminescence was measured for one hour for each sample. (d) represents the averages of three replicates for each concentration.
